# Biofertilizer and biostimulant properties of the microalga *Acutodesmus dimorphus*

**DOI:** 10.1007/s10811-015-0625-2

**Published:** 2015-05-29

**Authors:** Jesus Garcia-Gonzalez, Milton Sommerfeld

**Affiliations:** Department of Applied Sciences and Mathematics, College of Letters and Sciences, Arizona State University, Mesa, AZ 85212 USA; Arizona Center for Algae Technology and Innovation, Arizona State University, 7418 East Innovation Way South, ISTB-3, Mesa, AZ 85212 USA; Environmental and Resource Management Program, Ira A. Fulton Schools of Engineering, Polytechnic School, Mesa, AZ 85212 USA

**Keywords:** Biostimulant, Biofertilizer, Foliar spray, Microalgae, Seed primer, *Acutodesmus dimorphus*

## Abstract

Microalgae represent a potential sustainable alternative for the enhancement and protection of agricultural crops. Cellular extracts and dry biomass of the green alga *Acutodesmus dimorphus* were applied as a seed primer, foliar spray, and biofertilizer, to evaluate seed germination, plant growth, and fruit production in Roma tomato plants. *A. dimorphus* culture, culture growth medium, and different concentrations (0, 1, 5, 10, 25, 50, 75, and 100 %) of aqueous cell extracts in distilled water were used as seed primers to determine effects on germination. Seeds treated with *A. dimorphus* culture and with extract concentrations higher than 50 % (0.75 g mL^−1^) triggered faster seed germination—2 days earlier than the control group. The aqueous extracts were also applied as foliar fertilizers at various concentrations (0, 10, 25, 50, 75, and 100 %) on tomato plants. Extract foliar application at 50 % (3.75 g mL^−1^) concentration resulted in increased plant height and greater numbers of flowers and branches per plant. Two dry biomass treatments (50 and 100 g) were applied 22 days prior to seedling transplant and at the time of transplant to assess whether the timing of the biofertilizer application influenced the effectiveness of the biofertilizer. Biofertilizer treatments applied 22 days prior to seedling transplant enhanced plant growth, including greater numbers of branches and flowers, compared to the control group and the biofertilizer treatments applied at the time of transplant. The *A. dimorphus* culture, cellular extract, and dry biomass applied as a biostimulant, foliar spray, and biofertilizer, respectively, were able to trigger faster germination and enhance plant growth and floral production in Roma tomato plants.

## Introduction

In the coming decades, a crucial challenge will be meeting future food demands without causing further environmental degradation (Godfray, *et al.*^2010^; Odegard and van der Voet 2014). The expanding global population, and their anticipated adoption of calorie-rich diets heavily comprised of dairy and meat products, represent added pressure to the Earth’s resources. Society faces a challenge not only to increase agricultural production amidst global climate change, which threatens to diminish harvests in many areas of the world, but also to develop innovative technologies that increase agricultural yields, minimize inputs, and deter further environmental pollution (Tilman, *et al.*^2002^; Foley, *et al.*^2011^).

The overuse of synthetic agrochemicals has resulted in massive ecological degradation throughout the world, leading to ocean dead zones, eutrophication, soil infertility, and biodiversity loss (Köhler and Triebskorn 2013; Chagnon *et al.*^2014^; Hallmann, *et al.*^2014^; van der Sluijs *et al.*^2014^). The use of microalgae as biofertilizers provides a possible solution. Biofertilizers are considered to be an environmentally friendly, cost-effective, sustainable alternative to synthetic fertilizers, for they not only enhance agricultural production but also diminish environmental pollution (Kawalekar 2013). Biofertilizers are products that contain living microorganisms or natural compounds derived from organisms such as bacteria, fungi, and algae that improve soil chemical and biological properties, stimulate plant growth, and restore soil fertility (Abdel-Raouf, *et al.*^2012^).

Microalgae are multifunctional. They are capable of producing biomass that can be utilized for fuel, food, animal feed, and fertilizers (Metting 1990). Microalgae possess the potential to have a major influence on essential ecosystem services since they (i) can be cultivated in wastewater and agricultural runoff, recovering excess nutrients and reclaiming water for further use, and (ii) can sequester carbon dioxide and nitrous oxides from industrial sources, reducing greenhouse gas emissions (Brennan and Owende 2010). However, microalgae production must overcome several barriers in order for them to become economically viable, especially for production of biofuels (Brennan and Owende 2010; Mata, *et al.*^2010^; Wijffels and Barbosa 2010; Borowitzka 2013; Pragya, *et al.*^2013^).

One way to make microalgae biomass production more economically feasible, given current technologies, is to find potential applications for microalgae biomass or its by-products that enable producers to offset production costs. Given that microalgae contain high levels of micronutrients and macronutrients essential for plant growth, they have potential application as biofertilizers. Several studies have established an association between greater nutrient uptake, higher biomass accumulation, and greater crop yields to the incorporation of microalgae biofertilizers (Shaaban 2001; Faheed and Abd-El Fattah 2008).

A study investigating the effects of algae extracts on seed germination has observed faster germination and greater growth of rice seeds (Shukla and Gupta 1967). Other more recent studies obtained similar results utilizing seaweed extracts on tomato and wheat seeds, although they have also observed growth inhibition with increasing extract concentrations (Kumar and Sahoo 2011; Kumari, *et al.*^2011^; Hernández-Herrera, *et al*. 2013).

The cellular extracts and growth medium of several microalgae species have been shown to contain phytohormones (gibberellins, auxin, and cytokinin), which are known to play crucial roles in plant development (Tarakhovskaya, *et al*. 2007). Studies utilizing both the application of growth medium and cellular extracts from various algal species have shown a clear effect on plant development with the application of the algal extracts and the algal growth media (Burkiewicz 1987; Zhang, *et al.*^1989^; Tarakhovskaya, *et al.*^2007^; Stirk *et al.*^2013^; Grzesik and Romanowska-duda 2014). Similar studies utilizing seaweed extracts as foliar applied sprays have observed an increase in plant biomass accumulation and greater crop yields (Kumari *et al.*^2011^; Hernández-Herrera *et al.*^2013^).

The USA is the second largest tomato producer in the world, producing over 13 million tonnes in 2012 (FAOSTAT 2014), and utilizes vast quantities of fertilizers to maintain annual production rates. The objectives of this study were to investigate the potential agricultural applications of the robust green microalga *Acutodesmus dimorphus* as a seed primer, a foliar fertilizer, and a soil amendment or biofertilizer and assess its effects on seed germination and plant growth of Roma tomatoes (*Solanum lycopersicum* var. Roma) under greenhouse conditions.

## Materials and methods

### Cultivation and harvesting

The microalga *Acutodesmus dimorphus* (LARB-0414), isolated from the Phoenix, AZ, USA, metropolitan area, was cultivated outdoors in seven 1.22 m × 14.63 m production row panel photobioreactors using standard BG-11 algae culture medium (Stanier, *et al.*^1971^), bubbled with air mixed with 1 % carbon dioxide, at the Arizona State University, Arizona Center for Algae Technology and Innovation (AzCATI). The biomass was harvested by centrifugation at day 14 of cultivation and was then frozen until used.

The frozen biomass was thawed in a cold room at 4 °C for 24 h. Once thawed, the biomass was spread onto ten metal trays at a thickness of 1.5 cm and placed inside a freeze-dryer at −40 °C to freeze-dry for approximately 48 h. The dried biomass was then collected and stored in a cold room at 4 °C.

### Cell extracts

One kilogram of the freeze-dried biomass was suspended in distilled (DI) water at a concentration of 150 g L^−1^. The suspension was stirred on a stirring plate for 10 min to allow the biomass to dissociate. The suspension was then processed through a Microfluidizer (M-110EH-30)—a mobile high-shear fluid processor at a flow rate of 450 mL min^−1^ at 172 mPa to disrupt the cell wall and obtain the intracellular extracts. The resulting extract was then centrifuged at 8983×*g* for 10 min at 22 °C to separate the cell extracts from the biomass residue. To minimize potential degradation, the resulting extract supernatant was collected in a flask covered with aluminum foil and stored in a cold room at 4 °C. The biomass residue was also stored in the cold room for potential future use.

### Seed primer experiment

The cellular extracts, growth medium, and culture of *A. dimorphus* were screened to assess their ability to stimulate faster seed germination. The growth medium was obtained from a 14-day-old culture. *A. dimorphus* was cultivated indoors in a glass column photobioreactor (250 mL) using standard BG-11 algae culture medium (Stanier *et al.*^1971^), bubbled with air mixed with 1 % carbon dioxide. Two 50-mL culture samples were obtained by collecting the supernatant after centrifugation at 3724×*g* for 10 min.

Each treatment was replicated three times with ten seeds per replicate. The seeds were surface sterilized with 10 mL of 5 % solution of sodium hypochlorite for 10 min, rinsed twice with DI water, transferred to sterile Petri plates, and soaked in 10 mL of the respective treatment solutions for 24 h. Following the 24-h priming (soaking) period, the seeds were placed between two 42.5-mm Whatman no. 1 filter papers and allowed to dry for 24 h at room temperature (21 °C). Preceding the drying period, the seeds were transferred to a sterile 100-mm Petri plate containing a moist 75-mm Whatman no. 1 filter, which was soaked with 3 mL of DI water. The plates were incubated at room temperature at 21 °C under a 16-h light/8-h dark cycle.

Seed germination was checked at 24-h intervals over a period of 10 days and counted as germinated if at least 2 mm of the radicle had emerged. The filter paper for all treatments was saturated as needed with 3 mL of DI water to maintain moisture. Using a caliper, root, shoot, and leaf lengths (mm) were measured. Other measured variables included number of lateral roots, germination percentage (GP), and germination energy (GE). Germination percentage is an estimate of the viability of a population of seeds and was calculated as GP = (number of germinated seeds/total number of seeds) × 100. Germination energy, a measure of the speed of germination and assumed to be a measure of the vigor of the seedling produced, was calculated according to Hernández-Herrera et al. (2013), where GE = (number of germinating seeds/number of total seeds per treatment after germination for 3 days) × 100.

Treatments were as follows:

1. Control, 0 % extract (10 mL of DI water)
2. S_1_, 1 % concentration (0.1 mL extract in 9.9 mL DI water)
3. S_2_, 5 % concentration (0.5 mL extract in 9.5 mL DI water)
4. S_3_, 10 % concentration (1 mL extract in 9 mL DI water)
5. S_4_, 25 % concentration (2.5 mL extract in 7.5 mL DI water)
6. S_5_, 50 % concentration (5 mL extract in 5 mL DI water)
7. S_6_, 75 % concentration (7.5 mL extract in 2.5 mL DI water)
8. S_7_, 100 % concentration (10 mL)
9. S_8_, *Acutodesmus* growth medium (10 mL)
10. S_9_, *Acutodesmus* culture (10 mL)

### Foliar spray experiment

The experiment was performed under greenhouse conditions at approximately 28 ± 2 °C and 85 % relative humidity; treatments were arranged in a complete randomized block design. The experiment consisted of five treatments at various extract concentrations (0, 10, 25, 50, 75, and 100 %) diluted in distilled water (DI). Each treatment consisted of three replicates, one seedling per replicate. Each plant received two foliar applications; the first, at 50 mL, was applied at the time of transplant and the second, at 100 mL, 4 weeks later.

During foliar treatment applications, the soil surface was covered with aluminum foil to prevent spray runoff from coming in contact with the potting soil and being potentially available to be taken up by the roots. The sprays were conducted in the morning when the stomata were open due to water pressure, thus enabling greater foliar penetration. All plants were watered as needed throughout the experiment, except after foliar application when they were not watered for 24 h.

Treatments were as follows:

*For 50-mL spray treatments, the concentrations below were reduced in half to total 50 mL volume

1. Control, 0 % extract, 100 mL DI water
2. T_1_, 10 % (*v*/*v*) 10 mL extract in 90 mL DI water
3. T_2_, 25 % (*v*/*v*) 25 mL extract in 75 mL DI water
4. T_3_, 50 % (*v*/*v*) 50 mL extract in 50 mL DI water
5. T_4_, 75 % (*v*/*v*) 75 mL extract in 25 mL DI water
6. T_5_, 100 % (v/v) 100 mL extract

### Biofertilizer experiment

The biofertilizer experiment was conducted under greenhouse conditions at approximately 28 ± 2 °C, in 85 % relative humidity, from August through October 2014. Roma tomato (*S. lycopersicum* var. Roma) seeds were grown in sterilized potting soil, a mixture of vermiculite and peat moss. The seedlings were then transplanted after 22 days to 28-cm pots (one seedling per pot). Two biofertilizer treatments of 50 and 100 g of dry algal biomass were applied 22 days prior to seedling transplant, into pots containing potting soil (peat moss:vermiculite:perlite), mixed thoroughly, and watered once a week for 3 weeks prior to seedling transplant. The other two 50- and 100-g biofertilizer treatments were applied at the time of seedling transplant. Each treatment had three replicates and was set up in a completely randomized block design. Plants were grown for a total of 8 weeks and were hand watered as needed. Plant height (cm), number of flowers, number of branches, and early fruit development were recorded for all treatments. One sample per treatment was chosen at random to measure total fresh plant weight (g).

Treatments were as follows:

1. Control (no biofertilizer)
2. B_1_, 50 g of biofertilizer applied 22 days prior to transplant
3. B_2_, 100 g of biofertilizer applied 22 days prior to transplant
4. B_3_, 50 g of biofertilizer applied at the time of transplant
5. B_4_, 100 g of biofertilizer applied at the time of transplant

### Statistical analysis

Statistical analyses were conducted using StatPlus:mac LE programming (AnalystSoft Inc. 2009). All experiments were analyzed using a one-way analysis of variance (ANOVA), to test difference among the means. Post hoc *t* test was used for analysis of significant difference between treatments: the level of significance was set at *P* < 0.05.

## Results

### Biostimulant

**Fig. 1:**
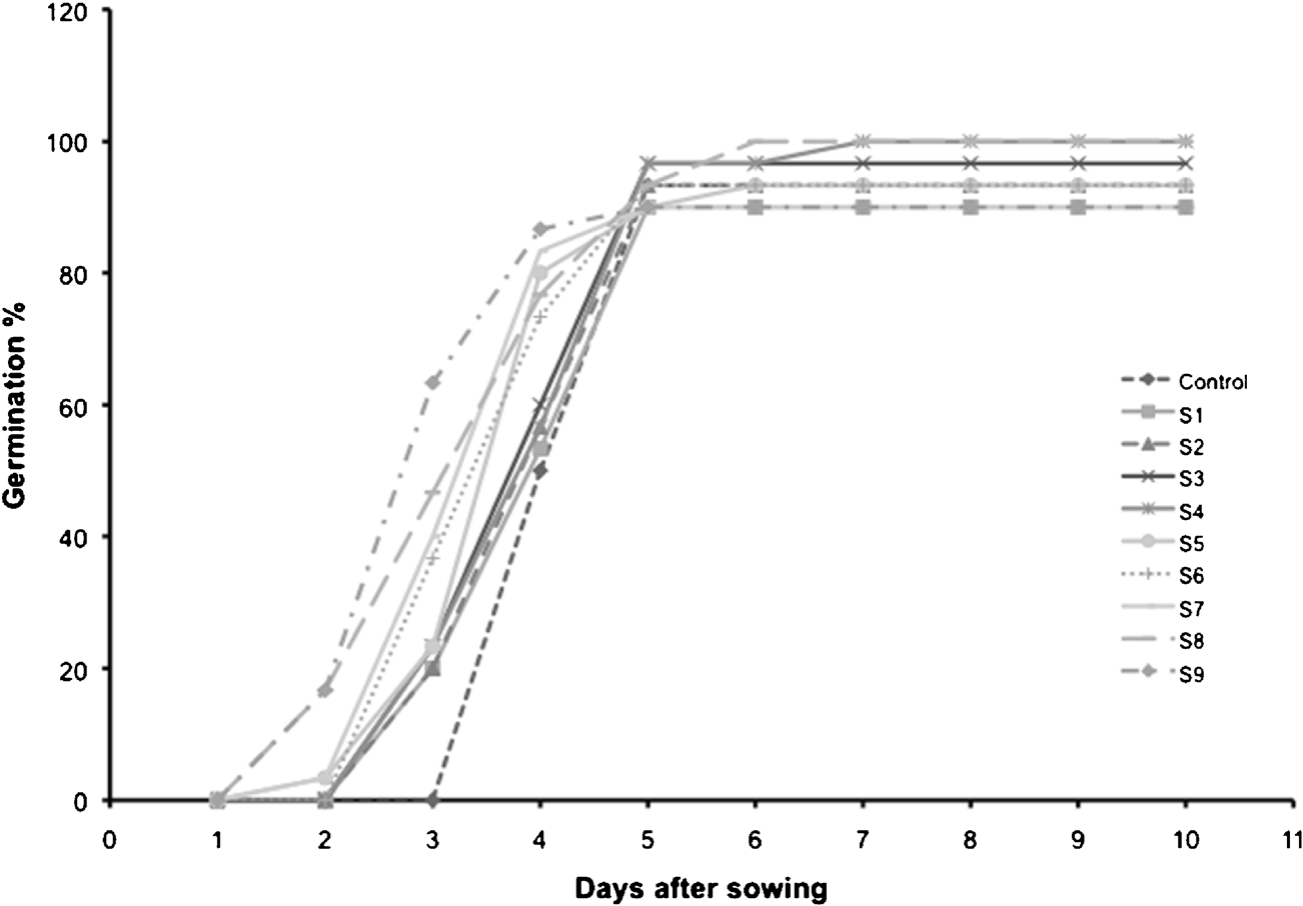
Seed germination percentage: seeds treated with *Acutodesmus* culture (S9), growth medium (S8), and cell extracts: *S1* 1 % extract concentration, *S2* 5 % extract concentration, *S3* 10 % extract concentration, *S4* 25 % extract concentration, *S5* 50 % extract concentration, *S6* 75 % extract concentration, and S7 100 % extract concentration. All extract dilutions were with DI water

The live *A. dimorphus* culture (S_9_), the filtered growth medium (S_8_), and 50 % (S_5_) and 100 % (S_7_) extract concentration treatments triggered faster seed germination—2 days earlier than the control group. The *A. dimorphus* culture treatment (S_9_) was the only treatment to have at least half of the seeds germinate by the third day. On the fifth day, most treatments had reached full germination percentage (Fig. 1). Almost all treatments, with the exception of the growth medium (S_8_) and the 25 % (0.375 g mL^−1^) extract concentration (S_4_), had seeds that did not germinate. Because the majority of the seeds within these treatments did germinate, it was concluded that some of the seeds were not viable.

**Fig. 2:**
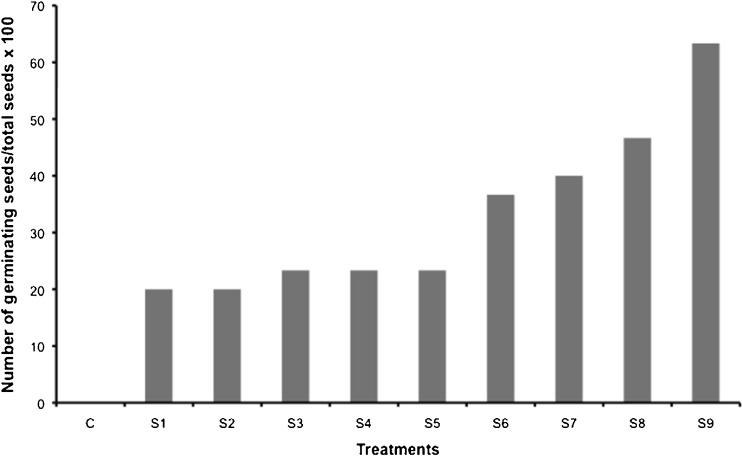
Germination energy of seeds treated with *Acutodesmus* culture (S9), growth medium (S8), and cell extracts: *S1* 1 % extract concentration, *S2* 5 % extract concentration, *S3* 10 % extract concentration, *S4* 25 % extract concentration, *S5* 50 % extract concentration, *S6* 75 % extract concentration, and *S7* 100 % extract concentration. All extract dilutions were with DI water

Germination energy calculations demonstrated a positive relationship between increasing extract concentrations and increasing speed of germination (germination energy). The greatest seed germination speed obtained with an extract treatment was with the 100 % (1.5 g mL^−1^) concentration (S_7_) at 40 %, slightly surpassed by the growth medium (S_8_) with 47 % germination energy. However, the fastest germination speed at 63 % was observed on seeds that were treated with *A. dimorphus* living culture (S_9_) (Fig. 2).

**Fig. 3:**
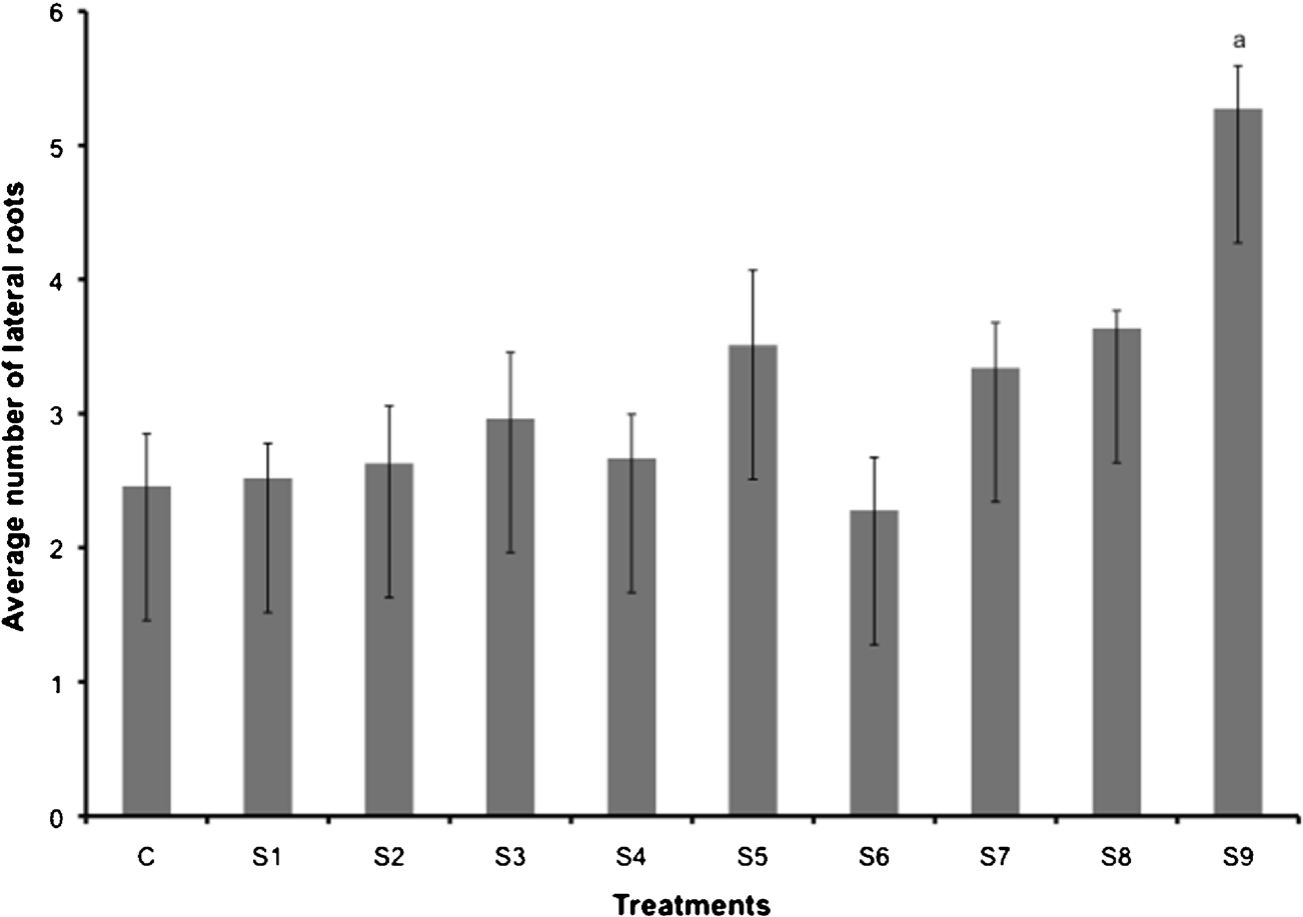
Effects of *Acutodesmus* culture (S9), growth medium (S8), and extract concentrations (*S1*–*S7*) on lateral root development of tomato seedlings. *S1* 1 % extract concentration, *S2* 5 % extract concentration, *S3* 10 % extract concentration, *S4* 25 % extract concentration, *S5* 50 % extract concentration, *S6* 75 % extract concentration, and *S7* 100 % extract concentration. All extract dilutions were with DI water. *Columns* denoted by a *different letter* are significantly different at *P* < 0.05. *Values* represent average (*n* = 10); *bars* represent standard error

The tomato seeds inoculated with *A. dimorphus* culture had greater lateral root development (Fig. 3) outperforming all other treatments. The number of lateral roots was somewhat variable across the extract concentration range. However, there was a general trend of more lateral roots with increasing extract concentration. The higher the number of lateral roots, the greater the plants’ ability to acquire water and nutrients: hence, seeds inoculated with *Acutodesmus* culture would potentially accumulate greater plant biomass and result in greater crop yields.

### Foliar spray

**Fig. 4:**
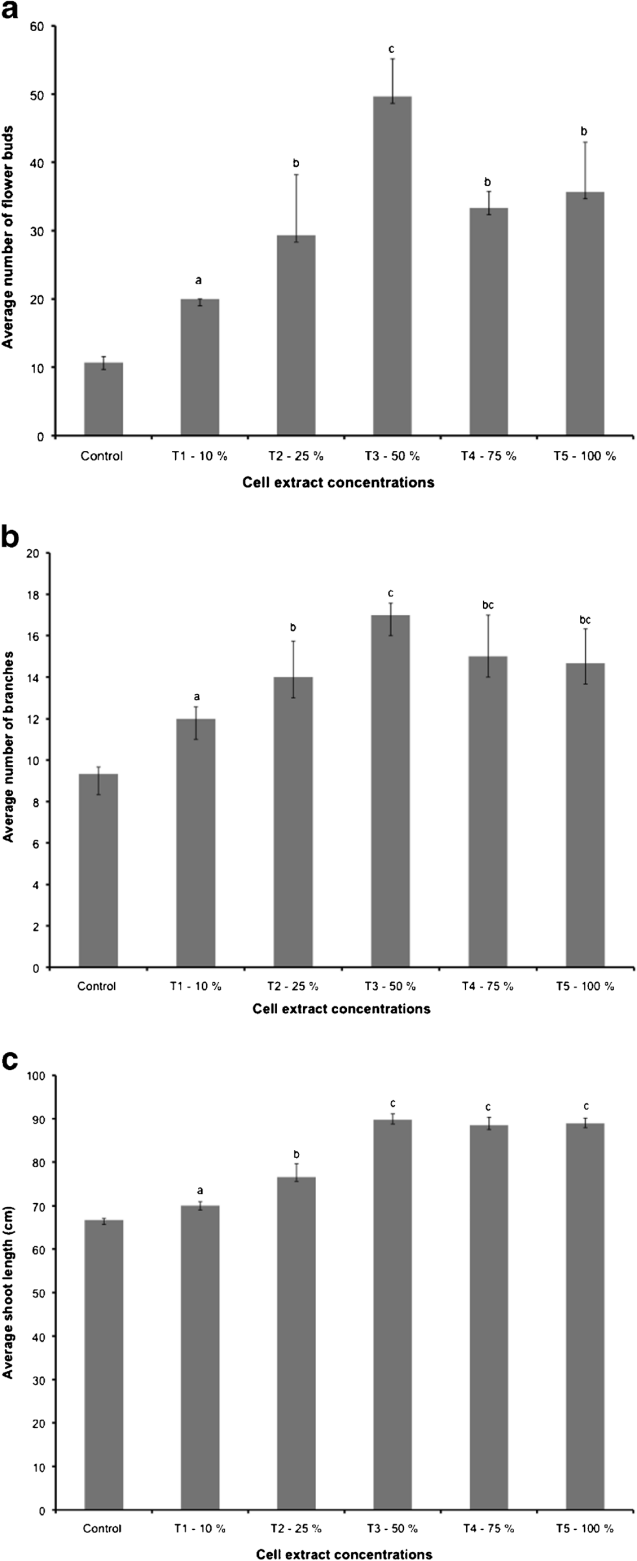
**a** Effects of cell extracts as foliar treatments on flower development, **b** effects of foliar treatments on average number of branches, **c** effects of foliar sprays on average shoot length per treatment on tomato plants: *T1* 10 % extract concentration, *T2* 25 % extract concentration, *T3* 50 % concentration, *T4* 75 % concentration, and *T5* 100 % extract concentration. All extract dilutions were with DI water. *Columns* denoted by a *different letter* are significantly different at *P* < 0.05. *Values* represent average (*n* = 3); *bars* represent standard error

All foliar treatments resulted in greater plant growth compared to the control group. The 50 % or 3.75 g mL^−1^ extract concentration (T_3_) foliar spray (7.5 g mL^−1^ for 100 mL second application) led to greater flower development (Fig. 4a), a higher number of branches (Fig. 4b), and the greatest plant height (Fig. 4c). Foliar sprays of higher concentrations (75 % (T_4_) and 100 % (T_5_)) resulted in less flower development, a lower number of branches, and a slight decrease in plant height compared to the 50 % extract concentration foliar treatment (Table 1).

**Table 1:** Effects of extract foliar sprays on growth parameters of tomato plants

Treatments	Concentrations (in DI water) (g mL^−1^)	Growth parameters		
		Number of flowers	Number of branches	Plant height (cm)
Control	0	10.67 ± 0.882	9.33 ± 0.333	66.68 ± 0.367
T_1_—10 %	0.75	20.00 ± 0.000	12 ± 0.577	70.06 ± 0.923
T_2_—25 %	1.875	29.33 ± 8.838	14 ± 1.732	76.62 ± 2.986
T_3_—50 %	3.75	49.67 ± 5.457	17 ± 0.577	89.75 ± 1.388
T_4_—75 %	5.625	33.33 ± 2.404	15 ± 2.000	88.48 ± 1.845
T_5_—100 %	7.5	35.67 ± 7.311	14.67 ± 1.667	88.90 ± 1.270

Values are average ± standard error (*n =* 3)

**Fig. 5:**
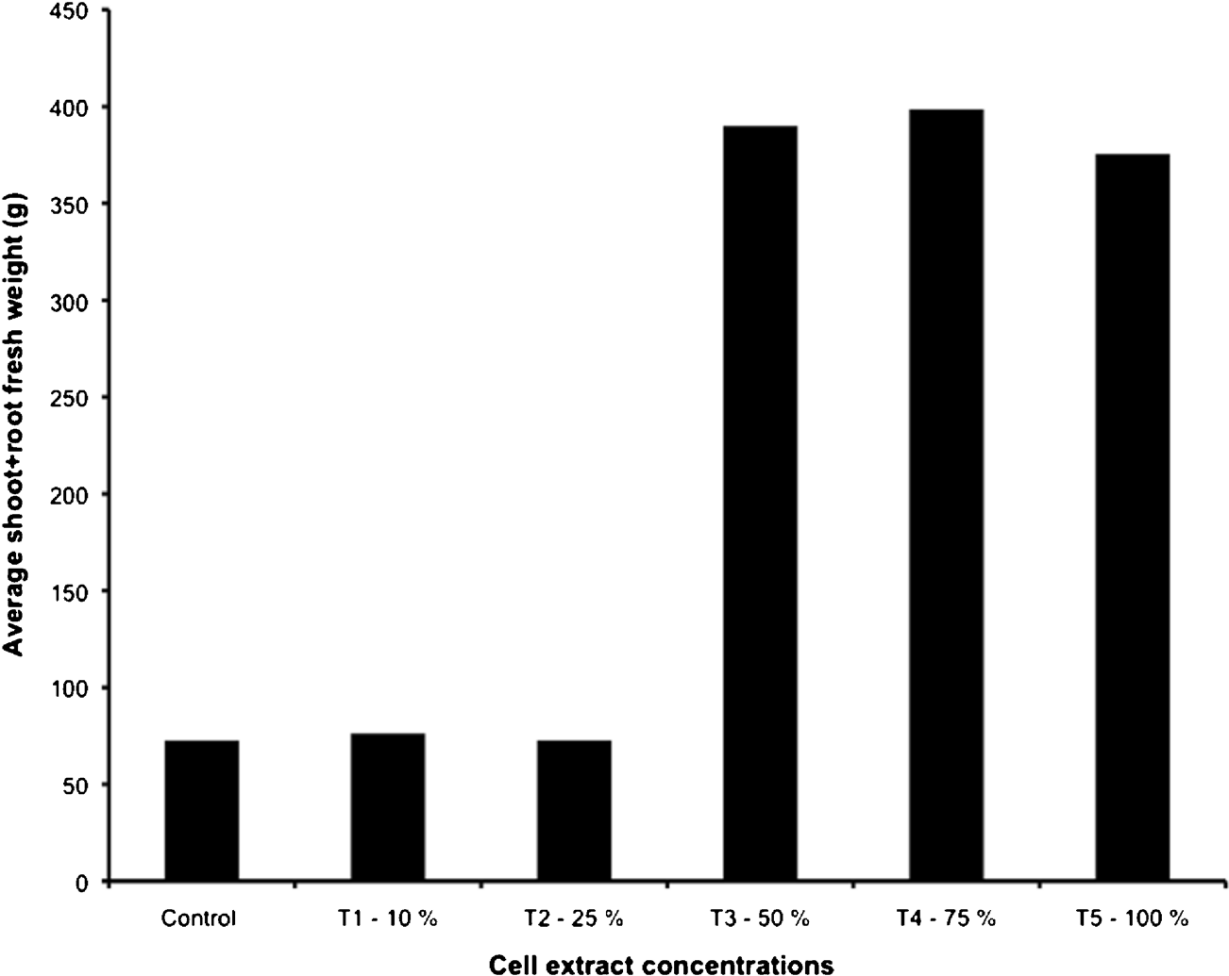
Effects of *Acutodesmus dimorphus* extracts as foliar sprays on total fresh plant weight (one sample per treatment chosen at random)

One plant sample per foliar treatment was chosen at random in order to measure the impact of foliar spray on total fresh plant weight (g). The 50 % or 3.75 g mL^−1^ treatment (T_3_), 75 % or 5.625 g mL^−1^ treatment (T_4_), and 100 % or 7.5 g mL^−1^ treatment (T_5_) extract concentrations led to ca. five times greater total plant fresh weight (Fig. 5). However, additional spray concentrations beyond the 50 % (3.75 g mL^−1^) did not show much additional increase in plant weight.

### Biofertilizer

**Fig. 6:**
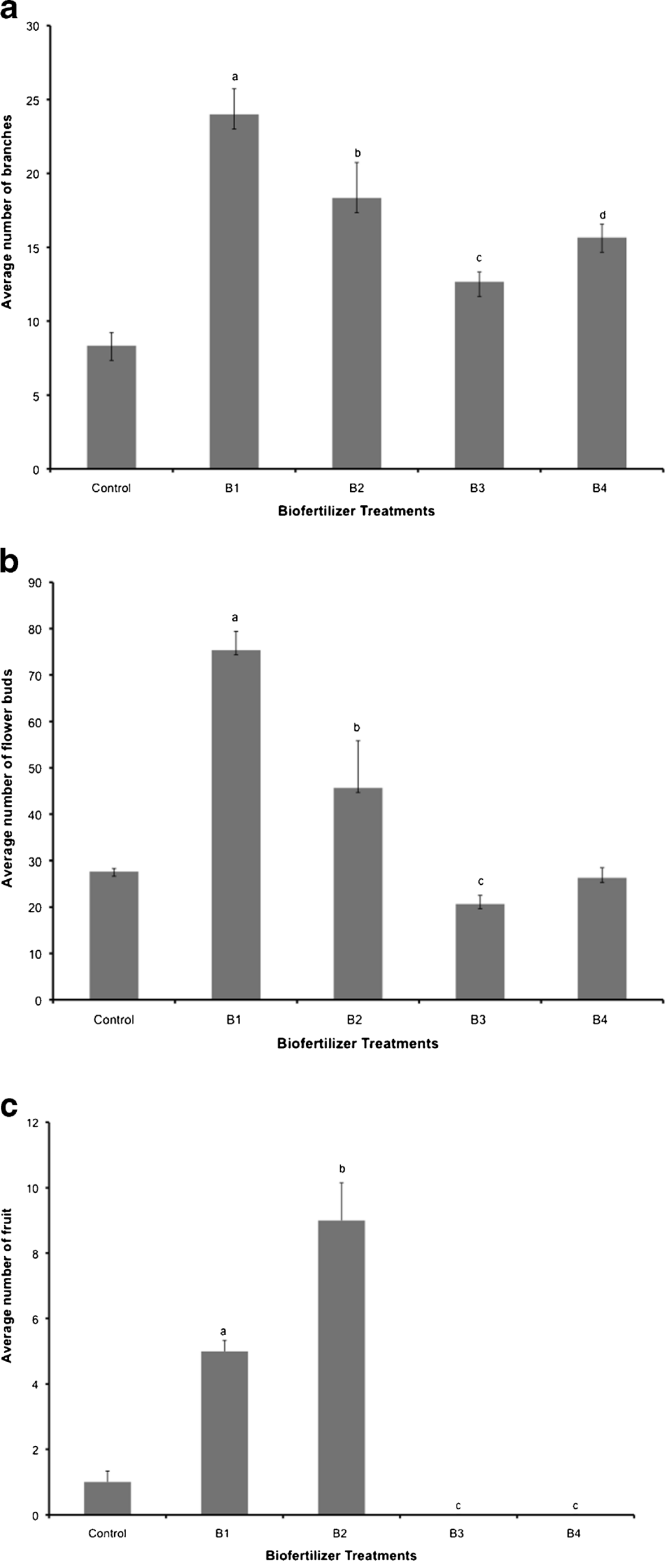
**a** Effects of biofertilizer on branch development—number of branches per plant (*P* = 0.0002), **b** effects on number of flowers (*P* = 0.0001), and **c** effects on early fruit setting—number of fruits (*P* = 0.0129): *B1* 50 g 22 days prior to transplant, *B2* 100 g 22 days prior to transplant, *B3* 50 g at the time of transplant, and *B4* 100 g at the time of transplant. *Columns* denoted by a *different letter* are significantly different at *P* < 0.05. *Values* represent average (*n* = 3); *bars* represent standard error

The results showed a significant difference between the biofertilizer treatments that were applied 22 days prior to seedling transplant (B_1_, B_2_) compared to those applied at the time of transplant (B_3_, B_4_). Differences in several important growth parameters, such as the number of branches (Fig. 6a), number of flowers (Fig. 6b), and early fruit development (Fig. 6c), were observed. There were significant differences when the biofertilizer treatments were applied 22 days prior to transplant as compared to the control group and the biofertilizer treatments applied at the time of transplant.

Focusing only on the biofertilizer treatments applied 22 days prior to transplant, it was apparent that the 50- and 100-g treatments or amendments (B_1_, B_2_) yielded a greater average number of branches and flowers than the control group. However, the lower amendment concentration gave greater numbers of fruit set. The opposite was observed when the biofertilizer treatments were applied during seedling transplant, with the 100-g biofertilizer treatment (B_4_) generating plants with a slightly greater number of branches and flowers than the 50-g treatment (B_3_) (Fig. 6a, b).

When comparing the biofertilizer treatments applied during seedling transplant (B_3_, B_4_) with the control group, we observed that for branch development (Fig. 6a) and flower development (Fig. 6b), the 100-g treatment exceeded the control group, but did not reach the levels observed with the biofertilizer treatments applied 22 days in advance. With flower development (Fig. 6b), the biofertilizer treatments gave results similar to those of the control group. The results on early fruit setting indicated that application of the biofertilizer 22 days in advance increased early fruit set with both 50- and 100-g treatments, and with the 100-g treatment showing the greater early fruit set. A very low fruit set (less than that of the control) occurred with the biofertilizer application (B_3_, B_4_) at the time of seedling transplant (Fig. 6c). This could possibly be due to nutrient imbalance, caused by microbial nutrient immobilization. It is also important to note that due to the early termination of the experiment, total fruit yields were not obtained. Nevertheless, given the early fruit setting data, we can presume that both biofertilizer treatments applied 22 days prior to seedling transplant would have yielded greater fruit numbers, with the 100-g biofertilizer treatment likely resulting in the highest yields.

**Fig. 7:**
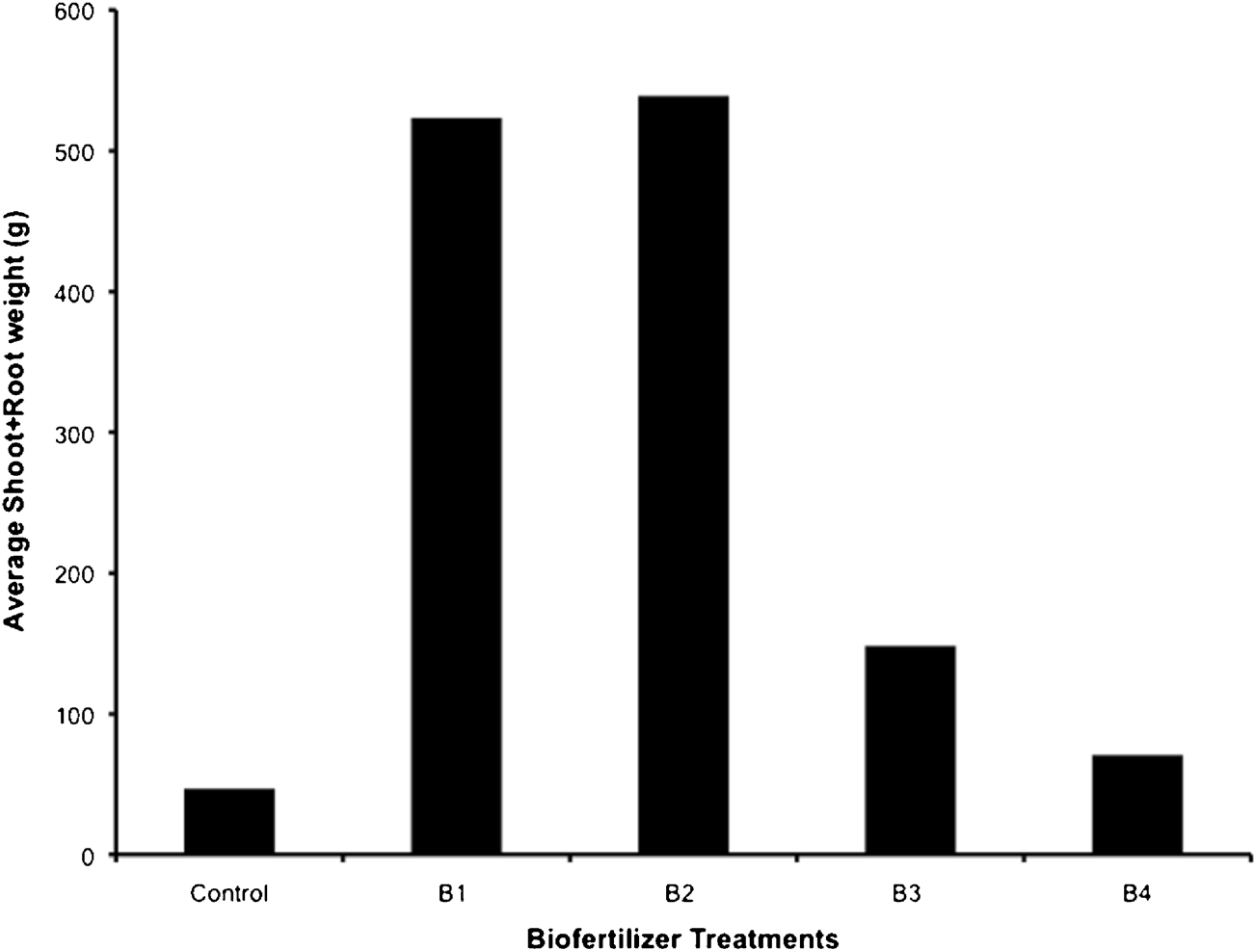
Total fresh plant (shoot + root) weight of samples selected at random from treatments: *B1* 50 g 22 days prior to transplant, *B2* 100 g 22 days prior to transplant, *B3* 50 g at the time of transplant, and *B4* 100 g at the time of transplant

General observations of the growing plants indicated differences in plant mass with the biofertilizer treatments. The results indicated that the biofertilizer treatments applied 22 days prior to seedling transplant (B_1_, B_2_) yielded significantly more biomass, having greater shoot and root fresh weights (Fig. 7). All biofertilized treatments led to higher total fresh weight compared to the control group. The two biofertilizer treatments applied 22 days prior to seedling transplant greatly exceeded the control group in biomass (400+ g difference) and weighed approximately 350 g more than the biofertilizer treatments applied at the time of seedling transplant. There was little difference in biomass between the two biofertilizer treatments applied 22 days prior to transplant, with the 100-g treatment (B_2_) showing a slightly greater total fresh plant weight than the 50-g treatment (B_1_), a difference of approximately 15 g.

## Discussion

The vast majority of research on the agricultural applications of algae has focused on the use of cyanobacteria (blue-green algae) on rice fields for various reasons, the most important being their ability to fix atmospheric nitrogen to plant-available forms (Irisarri, *et al.*^2001^; Jha and Prasad 2006; Pereira *et al.*^2008^; Sharma, *et al.*^2010^), or on macroalgae (seaweeds), since they can be harvested from coastal areas and are easier to process compared to microalgae (Verkleij 1992; Zodape 2001).

The renewed interest in developing biofuels from microalgae has led to an increase in research for potential product and/or by-product applications that can make cultivation and production more economically feasible at mass scales. Most of the research on the potential by-product applications of microalgae has concentrated on bioactive compounds (nutraceuticals, antioxidants, and food active ingredients) due to their high retail values (Herrero, *et al.*^2006^; Mendiola *et al.*^2007^; Plaza *et al.*^2010^; Rodríguez-Meizoso *et al.*^2010^). We believe that there is a larger untapped market for the application of microalgae biomass or by-products as agrochemicals.

There is limited evidence on the application of live microalgae culture, cell extracts, and dry biomass as potential agrochemicals. To increase our knowledge about the agricultural applications of microalgae, the goal of this study was to evaluate whether the microalga *A. dimorphus* living culture, cell extracts, and dry biomass could be applied to tomato plants as a biostimulant, foliar spray, and biofertilizer.

Previous studies have shown that certain microalgal extracts enhance the growth of agricultural crops, which has been attributed to plant growth regulators (auxins, gibberellins, and cytokinins) and to high levels of macro- and micronutrients (Tarakhovskaya, *et al.*^2007^). This study demonstrated that the application of *A. dimorphus* aqueous extracts, live culture, and dry biomass enhanced the germination, growth, and potential yield of tomato plants. Our study showed that the application of live *A. dimorphus* culture and aqueous cell extracts increased seed germination percentage in all treatments compared to the control group. The seeds treated with live *A. dimorphus* culture germinated faster, meaning the seedlings had the greatest vigor. Studies conducted on tomato seeds utilizing seaweed extracts at varying concentrations gave similar results. Hernández-Herrera et al. (2013) noticed that an extract concentration of 0.009 g mL^−1^ resulted in the highest germination percentage and the greatest plant growth, and that higher extract concentrations exhibited a negative effect on seed germination. Similarly, Kumar and Sahoo (2011) observed that seaweed extract concentrations greater than 20 % resulted in smaller root lengths, a lower number of lateral roots, and shorter shoot length.

Foliar application of the aqueous cell extracts of *A. dimorphus* increased plant growth compared to the control group, with the 50 % (3.75 g mL^−1^) extract concentration yielding the highest results. Foliar sprays of greater extract concentrations 75 and 100 % led to a decrease in growth compared to the 50 % spray. Our results are comparable to those obtained by Hernández-Herrera et al. (2013), who observed smaller shoot lengths on foliar sprays of seaweed extracts at concentrations greater than 0.18 g mL^−1^ (0.4 %), while Kumari et al. (2011) observed the opposite with greater plant growth with increasing seaweed extract concentrations.

Foliar sprays provide a more rapid nutrient utilization and enable faster correction of nutrient deficiencies compared to soil fertilizer applications. The greatest difficulty in supplying nutrients via foliar fertilization is in adequately applying the right quantity without damaging the leaves. Generally, the results have indicated a positive correlation between foliar extract applications and greater plant growth. Nevertheless, for foliar sprays to gain a greater acceptance for application in crop production, further studies need to be conducted since there are a plethora of factors (temperature, humidity, light intensity, nutrient concentration, surfactants, application rate, etc.) that may play a role in the efficiency of foliar applications (Haynes and Goh 1977; Kannan and Charnel 1986; Fernández and Eichert 2009).

In this study, tomato seedlings treated with dry *A. dimorphus* biomass (biofertilizer) clearly led to higher number of flowers and branches and early fruit development. There was a significant difference when the biomass was applied weeks prior to transplant, meaning that the biomass needs to be broken down in order to be readily available for plant uptake. Seedlings that received biofertilizer applications 22 days prior to transplant resulted in the greatest plant growth, the highest number of floral development, and early fruit setting, which could theoretically mean a greater crop yield. However, due to the early termination of our experiment, total crop yield data was not collected. Further studies should be conducted on utilizing whole and residual biomass (after oil extraction) to study whether it has the same stimulating growth effects, regardless of whether it is applied at the time of transplant or whether it needs to be applied earlier as seen with dry unprocessed (no oil extraction) biomass.

The application of live microalgae culture, aqueous cellular extracts, and dry biomass may not only increase plant growth but also make algae production systems more economically feasible. With increasing climate change, new innovations will be needed in order to enhance and protect agricultural crops throughout the world. The challenge to produce more food with limited resources makes microalgae a suitable alternative for enhancing and protecting agricultural production and delivering economic and environmental benefits to farmers and algae producers.

In conclusion, seed and foliar application of *A. dimorphus* aqueous extracts and growth medium had a major positive effect on seed germination and plant growth. However, as with synthetic agrochemicals, there appears to be a cutoff concentration at which higher extract concentrations lead to a decrease in the overall growth and development of tomato plants, compared to lower extract concentrations. Seed inoculation with living *A. dimorphus* culture had the greatest effect on seed germination and seedling growth compared to other treatments. Further studies on the applications of microalgae culture, growth medium, and cell extracts on different plant species are needed. This study demonstrated that there are positive correlations between aqueous foliar sprays and growth of tomato plants.

Application of dry *A. dimorphus* biomass as a biofertilizer for tomato plants led to an increase in plant growth. Earlier biofertilizer application (22 days prior to transplant) significantly enhanced plant growth, suggesting that earlier application is necessary for the biomass to be broken down in order for nutrients to become available for plant uptake. An application of 50 g of biofertilizer per plant was sufficient to be both productive and to be potentially feasible from an economic standpoint. Further studies should be conducted to observe how the *A. dimorphus* biomass will react under actual field conditions.

Additional studies on floral crops should be conducted since foliar sprays are commonly utilized within the floriculture industry. Microalgae are an enormous untapped resource with great potential in the agriculture sector, and additional research should be conducted to discover and exploit their potential.
